# Supplementary material for: Evolution of the highest fidelity DNA replication systems
Source: bioRxiv. 2026 Jun 2:2026.04.16.719065. Originally published 2026 Apr 17. Preprint. [Version 2] doi: 10.64898/2026.04.16.719065 (PMC13104929; doi:10.64898/2026.04.16.719065)
Supplement: Supplement 1 [file media-1.pdf]

# Supplementary Information: Evolution of the highest fidelity DNA replication systems

Stephan Baehr, Cooper Call

April 11, 2026

Table 1: Biological Parameters Across Diverse Species

| Species | Measurement Type | Reference |
|---|---|---|
| **D. pulex** | mutation rate | Lynch et al. (2023) |
| | age first reproduction | Lynch (1984) |
| | age last reproduction | Lynch (1984) |
| | generation time | N/A |
| | temperature | Keith et al. (2016) |
| | # of cell divisions | N/A |
| | dry mass | Lynch et al. (2023) |
| | genome size | Lynch et al. (2023) |
| **D. magna** | mutation rate | Lynch et al. (2023) |
| | age first reproduction | Lynch (1984) |
| | age last reproduction | Lynch (1984) |
| | generation time | N/A |
| | temperature | N/A |
| | # of cell divisions | N/A |
| | dry mass | Lynch et al. (2023) |
| | genome size | Lynch et al. (2023) |
| **S. cerevisiae** | mutation rate | Lynch et al. (2023) |
| | age first reproduction | Sherman (2002) |
| | age last reproduction | Minois et al. (2005) |
| | generation time | N/A |
| | temperature | Parapouli et al. (2020) |
| | # of cell divisions | N/A |
| | dry mass | Lynch et al. (2023) |
| | genome size | Lynch et al. (2023) |
| **H. sapiens** | mutation rate | Lynch et al. (2023) |
| | age first reproduction | N/A |
| | age last reproduction | N/A |
| | generation time | Wang et al. (2023) |
| | temperature | Geneva et al. (2019) |
| | # of cell divisions | Drost and Lee (1995) |
| | dry mass | Lynch et al. (2023) |
| | genome size | Lynch et al. (2023) |
| **M. musculus** | mutation rate | Lynch et al. (2023) |
| | age first reproduction | De Magalhães and Costa (2009) |

**Table 1 – continued from previous page**

| **Species** | **Measurement Type** | **Reference** |
|---|---|---|
| | age last reproduction | De Magalhães and Costa (2009) |
| | generation time | N/A |
| | temperature | Mei et al. (2018) |
| | # of cell divisions | Drost and Lee (1995) |
| | dry mass | Lynch et al. (2023) |
| | genome size | Lynch et al. (2023) |
| **C. elegans** | mutation rate | Lynch et al. (2023) |
| | age first reproduction | Zhang et al. (2020) |
| | age last reproduction | Zhang et al. (2020) |
| | generation time | N/A |
| | temperature | Flavel et al. (2018) |
| | # of cell divisions | Denver et al. (2009) |
| | dry mass | Lynch et al. (2023) |
| | genome size | Lynch et al. (2023) |
| **C. lupus** | mutation rate | Lynch et al. (2023) |
| | age first reproduction | N/A |
| | age last reproduction | N/A |
| | generation time | Mech et al. (2016) |
| | temperature | Kreeger et al. (1990) |
| | # of cell divisions | N/A |
| | dry mass | Lynch et al. (2023) |
| | genome size | Lynch et al. (2023) |
| **F. catus** | mutation rate | Lynch et al. (2023) |
| | age first reproduction | De Magalhães and Costa (2009) |
| | age last reproduction | De Magalhães and Costa (2009) |
| | generation time | N/A |
| | temperature | Giannetto et al. (2022) |
| | # of cell divisions | N/A |
| | dry mass | Lynch et al. (2023) |
| | genome size | Lynch et al. (2023) |
| **E. coli** | mutation rate | Lynch et al. (2023) |
| | age first reproduction | Gibson et al. (2018) |
| | age last reproduction | Wang et al. (2010) |
| | generation time | N/A |
| | temperature | Lee et al. (2012) |
| | # of cell divisions | N/A |
| | dry mass | Lynch et al. (2023) |
| | genome size | Lynch et al. (2023) |
| **C. crescentus** | mutation rate | Lynch et al. (2023) |
| | age first reproduction | Wright et al. (2015) |
| | age last reproduction | Ackermann et al. (2003) |
| | generation time | N/A |
| | temperature | Long et al. (2018) |
| | # of cell divisions | N/A |
| | dry mass | Lynch et al. (2023) |
| | genome size | Lynch et al. (2023) |

**Table 1 – continued from previous page**

| Species | Measurement Type | Reference |
|---|---|---|
| **P. tetraurelia** | mutation rate | Lynch et al. (2023) |
| | age first reproduction | Sung et al. (2012) |
| | age last reproduction | Takagi et al. (1987) |
| | generation time | N/A |
| | temperature | Luhring and DeLong (2016) |
| | # of cell divisions | N/A |
| | dry mass | Lynch et al. (2023) |
| | genome size | Lynch et al. (2023) |
| **T. thermophila** | mutation rate | Lynch et al. (2023) |
| | age first reproduction | Seyfert et al. (1984) |
| | age last reproduction | Brito et al. (2010) |
| | generation time | N/A |
| | temperature | Long et al. (2013) |
| | # of cell divisions | N/A |
| | dry mass | Lynch et al. (2023) |
| | genome size | Lynch et al. (2023) |
| **P. troglodytes** | mutation rate | Lynch et al. (2023) |
| | age first reproduction | De Magalhães and Costa (2009) |
| | age last reproduction | De Magalhães and Costa (2009) |
| | generation time | N/A |
| | temperature | Jensen et al. (2009) |
| | # of cell divisions | N/A |
| | dry mass | Lynch et al. (2023) |
| | genome size | Lynch et al. (2023) |
| **D. melanogaster** | mutation rate | Lynch et al. (2023) |
| | age first reproduction | Fernández-Moreno et al. (2007) |
| | age last reproduction | Piper and Partridge (2016) |
| | generation time | N/A |
| | temperature | Mołoń et al. (2020) |
| | # of cell divisions | Drost and Lee (1995) |
| | dry mass | Lynch et al. (2023) |
| | genome size | Lynch et al. (2023) |
| **A. thaliana** | mutation rate | Lynch et al. (2023) |
| | age first reproduction | Boyes et al. (2001) |
| | age last reproduction | Boyes et al. (2001) |
| | generation time | N/A |
| | temperature | Rivero et al. (2014) |
| | # of cell divisions | N/A |
| | dry mass | Lynch et al. (2023) |
| | genome size | Lynch et al. (2023) |
| **B. taurus** | mutation rate | Lynch et al. (2023) |
| | age first reproduction | De Magalhães and Costa (2009) |
| | age last reproduction | De Magalhães and Costa (2009) |

**Table 1 – continued from previous page**

| Species | Measurement Type | Reference |
|---|---|---|
| | generation time | N.A |
| | temperature | Sammes et al. (2019) |
| | # of cell divisions | N/A |
| | dry mass | Lynch et al. (2023) |
| | genome size | Lynch et al. (2023) |
| **P. sitchensis** | mutation rate | Lynch et al. (2023) |
| | age first reproduction | Trojan et al. (2026) |
| | age last reproduction | Trojan et al. (2026) |
| | generation time | N/A |
| | temperature | N/A |
| | # of cell divisions | N/A |
| | dry mass | Lynch et al. (2023) |
| | genome size | Lynch et al. (2023) |
| **S. pombe** | mutation rate | Lynch et al. (2023) |
| | age first reproduction | Vyas et al. (2021) |
| | age last reproduction | Spivey et al. (2017) |
| | generation time | N/A |
| | temperature | Farlow et al. (2015) |
| | # of cell divisions | N/A |
| | dry mass | Lynch et al. (2023) |
| | genome size | Lynch et al. (2023) |
| **B. mysticetus** | mutation rate | Lynch et al. (2023) |
| | age first reproduction | George et al. (2021b) |
| | age last reproduction | Breed et al. (2024) |
| | generation time | N/A |
| | temperature | George et al. (2021a) |
| | # of cell divisions | N/A |
| | dry mass | Lynch et al. (2023) |
| | genome size | Lynch et al. (2023) |

# References


Ackermann, M., Stearns, S. C. and Jenal, U. (2003). Senescence in a Bacterium with Asymmetric Division. Science *300*, 1920–1920.

Boyes, D. C., Zayed, A. M., Ascenzi, R., McCaskill, A. J., Hoffman, N. E., Davis, K. R. and Görlach, J. (2001). Growth Stage–Based Phenotypic Analysis of Arabidopsis: A Model for High Throughput Functional Genomics in Plants. The Plant Cell *13*, 1499–1510.

Breed, G. A., Vermeulen, E. and Corkeron, P. (2024). Extreme longevity may be the rule not the exception in Balaenid whales. Science Advances *10*, eadq3086.

Brito, P. H., Guilherme, E., Soares, H. and Gordo, I. (2010). Mutation accumulation in Tetrahymena. BMC evolutionary biology *10*, 354.

De Magalhães, J. P. and Costa, J. (2009). A database of vertebrate longevity records and their relation to other life-history traits. Journal of Evolutionary Biology *22*, 1770–1774.

Denver, D. R., Dolan, P. C., Wilhelm, L. J., Sung, W., Lucas-Lledó, J. I., Howe, D. K., Lewis, S. C., Okamoto, K., Thomas, W. K., Lynch, M. and Baer, C. F. (2009). A genome-wide view of

*Caenorhabditis elegans* base-substitution mutation processes. Proceedings of the National Academy of Sciences *106*, 16310–16314.

Drost, J. B. and Lee, W. R. (1995). Biological basis of germline mutation: comparisons of spontaneous germline mutation rates among drosophila, mouse, and human. Environmental and Molecular Mutagenesis *25 Suppl 26*, 48–64.

Farlow, A., Long, H., Arnoux, S., Sung, W., Doak, T. G., Nordborg, M. and Lynch, M. (2015). The Spontaneous Mutation Rate in the Fission Yeast *Schizosaccharomyces pombe*. Genetics *201*, 737–744.

Fernández-Moreno, M. A., Farr, C. L., Kaguni, L. S. and Garesse, R. (2007). Drosophila melanogaster as a Model System to Study Mitochondrial Biology. In Mitochondria, (Walker, J. M., Leister, D. and Herrmann, J. M., eds), vol. 372, pp. 33–49. Humana Press Totowa, NJ. Series Title: Methods in Molecular Biology.

Flavel, M. R., Mechler, A., Shahmiri, M., Mathews, E. R., Franks, A. E., Chen, W., Zanker, D., Xian, B., Gao, S., Luo, J., Tegegne, S., Doneski, C. and Jois, M. (2018). Growth of *Caenorhabditis elegans* in Defined Media Is Dependent on Presence of Particulate Matter. G3 Genes|Genomes|Genetics *8*, 567–575.

Geneva, I. I., Cuzzo, B., Fazili, T. and Javaid, W. (2019). Normal Body Temperature: A Systematic Review. Open Forum Infectious Diseases *6*, ofz032.

George, J., Horstmann, L., Fortune, S., Sformo, T. L., Elsner, R. and Follmann, E. (2021a). Chapter 16 - Thermoregulation and energetics. In The Bowhead Whale, (George, J. and Thewissen, J., eds), pp. 237–260. Academic Press.

George, J., Thewissen, J., Von Duyke, A., Breed, G. A., Suydam, R., Sformo, T. L., Person, B. T. and Brower, H. (2021b). Life history, growth, and form. In The Bowhead Whale pp. 87–115. Elsevier.

Giannetto, C., Acri, G., Pennisi, M., Piccione, G., Arfuso, F., Falcone, A., Giudice, E. and Di Pietro, S. (2022). Short Communication: Use of Infrared Thermometers for Cutaneous Temperature Recording: Agreement with the Rectal Temperature in Felis catus. Animals: an open access journal from MDPI *12*, 1275.

Gibson, B., Wilson, D. J., Feil, E. and Eyre-Walker, A. (2018). The distribution of bacterial doubling times in the wild. Proceedings. Biological Sciences *285*, 20180789.

Jensen, S. A., Mundry, R., Nunn, C. L., Boesch, C. and Leendertz, F. H. (2009). Non-invasive Body Temperature Measurement of Wild Chimpanzees Using Fecal Temperature Decline. Journal of Wildlife Diseases *45*, 542–546.

Keith, N., Tucker, A. E., Jackson, C. E., Sung, W., Lucas Lledó, J. I., Schrider, D. R., Schaack, S., Dudycha, J. L., Ackerman, M., Younge, A. J., Shaw, J. R. and Lynch, M. (2016). High mutational rates of large-scale duplication and deletion in *Daphnia pulex*. Genome Research *26*, 60–69.

Kreeger, T. J., Kuechle, V. B., Mech, L. D., Tester, J. R. and Seal, U. S. (1990). Physiological Monitoring of Gray Wolves (Canis lupus) by Radiotelemetry. Journal of Mammalogy *71*, 258–261.

Lee, H., Popodi, E., Tang, H. and Foster, P. L. (2012). Rate and molecular spectrum of spontaneous mutations in the bacterium *Escherichia coli* as determined by whole-genome sequencing. Proceedings of the National Academy of Sciences *109*.

Long, H., Sung, W., Kucukyildirim, S., Williams, E., Miller, S. F., Guo, W., Patterson, C., Gregory, C., Strauss, C., Stone, C., Berne, C., Kysela, D., Shoemaker, W. R., Muscarella, M. E., Luo, H., Lennon, J. T., Brun, Y. V. and Lynch, M. (2018). Evolutionary determinants of genome-wide nucleotide composition. Nature Ecology & Evolution *2*, 237–240.

Long, H.-A., Paixão, T., Azevedo, R. B. R. and Zufall, R. A. (2013). Accumulation of Spontaneous Mutations in the Ciliate *Tetrahymena thermophila*. Genetics *195*, 527–540.

Luhring, T. M. and DeLong, J. P. (2016). Predation changes the shape of thermal performance curves for population growth rate. Current Zoology *62*, 501–505.

Lynch, M. (1984). The Limits to Life History Evolution in Daphnia. Evolution *38*, 465–482.

Lynch, M., Ali, F., Lin, T., Wang, Y., Ni, J. and Long, H. (2023). The divergence of mutation rates and spectra across the Tree of Life. EMBO reports *24*, e57561.

Mech, L. D., Barber-Meyer, S. M. and Erb, J. (2016). Wolf (Canis lupus) Generation Time and Proportion of Current Breeding Females by Age. PLOS ONE *11*, e0156682.

Mei, J., Riedel, N., Grittner, U., Endres, M., Banneke, S. and Emmrich, J. V. (2018). Body temperature measurement in mice during acute illness: implantable temperature transponder versus surface infrared thermometry. Scientific Reports *8*, 3526.

Minois, N., Frajnt, M., Wilson, C. and Vaupel, J. W. (2005). Advances in measuring lifespan in the yeast Saccharomyces cerevisiae. Proceedings of the National Academy of Sciences of the United States of America *102*, 402–406.

Mołoń, M., Dampc, J., Kula-Maximenko, M., Zebrowski, J., Mołoń, A., Dobler, R., Durak, R. and Skoczowski, A. (2020). Effects of Temperature on Lifespan of Drosophila melanogaster from Different Genetic Backgrounds: Links between Metabolic Rate and Longevity. Insects *11*, 470.

Parapouli, M., Vasileiadis, A., Afendra, A.-S. and Hatziloukas, E. (2020). Saccharomyces cerevisiae and its industrial applications. AIMS microbiology *6*, 1–31.

Piper, M. D. W. and Partridge, L. (2016). Protocols to Study Aging in Drosophila. In Drosophila, (Dahmann, C., ed.), vol. 1478, pp. 291–302. Springer New York New York, NY. Series Title: Methods in Molecular Biology.

Rivero, L., Scholl, R., Holomuzki, N., Crist, D., Grotewold, E. and Brkljacic, J. (2014). Handling Arabidopsis Plants: Growth, Preservation of Seeds, Transformation, and Genetic Crosses. In Arabidopsis Protocols, (Sanchez-Serrano, J. J. and Salinas, J., eds), vol. 1062, pp. 3–25. Humana Press Totowa, NJ. Series Title: Methods in Molecular Biology.

Sammes, S. L., Lees, A. M., Cawdell-Smith, J. A. and Gaughan, J. B. (2019). 147 Changes in body temperature of lot-fed Bos taurus and Bos indicus steers during a heat wave. Journal of Animal Science *97*, 150–150.

Seyfert, H. M., Hipke, H. and Schmidt, W. (1984). Isolation And Phenotypic Characterization Of *Tetrahymena Thermophila* Size Mutants: The Relationship Between Cell Size And Regulation Of Dna Content. Journal of Cell Science *67*, 203–215.

Sherman, F. (2002). Getting started with yeast. In Methods in Enzymology vol. 350, pp. 3–41. Elsevier.

Spivey, E. C., Jones, S. K., Rybarski, J. R., Saifuddin, F. A. and Finkelstein, I. J. (2017). An aging-independent replicative lifespan in a symmetrically dividing eukaryote. eLife *6*, e20340.

Sung, W., Tucker, A. E., Doak, T. G., Choi, E., Thomas, W. K. and Lynch, M. (2012). Extraordinary genome stability in the ciliate *Paramecium tetraurelia*. Proceedings of the National Academy of Sciences *109*, 19339–19344.

Takagi, Y., Nobuoka, T. and Doi, M. (1987). Clonal lifespan of *Paramecium tetraurelia:* Effect of selection on its extension and use of fissions for its determination. Journal of Cell Science *88*, 129–138.

Trojan, V., Vacek, Z., Vacek, S., Tomczak, K., Černý, J., Cukor, J., Zeidler, A., Gallo, J. and Brabec, P. (2026). A global review of Sitka spruce (Picea sitchensis [Bong.] Carr.): challenges and opportunities in European modern forest management. European Journal of Forest Research *145*, 55.

Vyas, A., Freitas, A. V., Ralston, Z. A. and Tang, Z. (2021). Fission Yeast Schizosaccharomyces pombe: A Unicellular "Micromammal" Model Organism. Current Protocols *1*, e151.

Wang, P., Robert, L., Pelletier, J., Dang, W. L., Taddei, F., Wright, A. and Jun, S. (2010). Robust growth of Escherichia coli. Current biology: CB *20*, 1099–1103.

Wang, R. J., Al-Saffar, S. I., Rogers, J. and Hahn, M. W. (2023). Human generation times across the past 250,000 years. Science Advances *9*, eabm7047.

Wright, C. S., Banerjee, S., Iyer-Biswas, S., Crosson, S., Dinner, A. R. and Scherer, N. F. (2015). Intergenerational continuity of cell shape dynamics in Caulobacter crescentus. Scientific Reports *5*, 9155.

Zhang, S., Li, F., Zhou, T., Wang, G. and Li, Z. (2020). Caenorhabditis elegans as a Useful Model for Studying Aging Mutations. Frontiers in Endocrinology *11*, 554994.
